# The Increase in Frequency of Protective Behavior against Pesticide Poisoning in Narail, Bangladesh through Use of an Easy Paper Checklist; an Interventional Study

**DOI:** 10.3390/ijerph18179349

**Published:** 2021-09-04

**Authors:** Yurie Kobashi, Syed Emdadul Haque, Yoshitaka Nishikawa, Tomohiro Morita, Hiroshi Nagami, Kayako Sakisaka, Sanzida Mubassara, Masaharu Tsubokura

**Affiliations:** 1 Department of General Internal Medicine, Hirata Central Hospital, Hirata 963-8202, Japan; yoshitakanishikawa@gmail.com (Y.N.); tsubokura-tky@umin.ac.jp (M.T.); 2 UChicago Research Bangladesh, Dhaka 1230, Bangladesh; emdad91@gmail.com; 3 Department of General Internal Medicine, Soma Central Hospital, Soma 976-0016, Japan; t.morita526@gmail.com; 4 Institute for Prevention of Pesticide Adverse Effect on Human, Yamatokoriyama 639-1001, Japan; nagami@dia.janis.or.jp; 5 Teikyo University Graduate School of Public Health, Itabash, Tokyo 173-8605, Japan; sakisaka@med.teikyo-u.ac.jp; 6 Department of Botany, Jahangirnagar University, Savar Union 1342, Bangladesh; sanzida.botany@gmail.com

**Keywords:** farmworker, checklist, interventional study, occupational health

## Abstract

Protecting the health of farmworkers is a crucial issue. Previous studies report that safety training and educational interventions might increase farmworkers’ protective behaviors. The present study aimed to investigate the effectiveness of distributing a checklist as an interventional measure for pesticide protection in rural Asia, where pesticide poisoning is a major problem. This study was a community-based interventional study, using the distribution of a checklist with pesticide protective habits in Narail district, Bangladesh, with a total of 100 eligible males. Two questionnaire surveys were conducted before distributing the checklist and 25 days after. Change between the baseline and follow-up surveys was measured by frequency scores of protective behavior. The average pesticide-protective behavioral score increased from 4.58 in the baseline survey to 8.11 in the follow-up. Additionally, the checklist was more effective in the group with higher education, the younger group, and the group with lower pesticide-protective behavioral scores in the baseline survey. The paper checklist on protective behaviors against pesticide poisoning was effective because of the increase in the frequency of such positive behavior among farmworkers. Thus, intervention measures should be implemented to increase the knowledge and awareness regarding pesticide protection habits to protect the health of farmworkers.

## 1. Introduction

Vulnerable persons are defined as those with disease, disability, or related personal factors in poor societal or environmental conditions or in vulnerable circumstances because of social or legal stigmatization associated with their activity or identity. However, the definition has been slightly flexible in the context of this research [1]. Improving the health status of vulnerable populations is a crucial part of delivering essential health care to all [2] and achieving the sustainable development goals as laid out by the United Nations. In particular, agricultural workers represent a potentially vulnerable population due to a combination of unique social and cultural risk factors, including exposure to inherent hazards, being of lower economic status, living in rural areas, or having low educational status [3]. Additionally, it has been reported that pesticide exposure among agricultural workers was linked to specific health problems, DNA damage, neurological disorders, and respiratory effects [3,4,5,6]. Thus, protecting agricultural workers’ health is a crucial issue in public health matters.

For sustaining health among agricultural workers, various efforts have been undertaken. In Asian developing countries, the majority of the rural population is engaged in agricultural work, and pesticide poisoning is a significant health problem among rural farmworkers [7,8]. In addition, farmworkers frequently lack adequate knowledge about the occupational hazards they face and the relevant risk factors [9]. In this context, previous studies have indicated that safety training and educational interventions might increase protective behaviors [7,10,11,12,13]. Nevertheless, the evidence of easy and effective interventional measures for pesticide protection is limited, especially for rural Asia [10].

Bangladesh, classified as a low-middle-income country, is located in Southeast Asia. A total of 5.3% of residents engage in agricultural work in Bangladesh, of whom approximately 58% are from the lowest wealth quantile and approximately 93% work in a rural area [14]. In this context, pesticide poisoning is a major health problem in Bangladesh and is especially associated with suicide [15]. Consequently, the government has banned highly hazardous pesticides for agricultural use, and these measures have reduced the mortality and hospitalization rates in terms of pesticide poisoning; however, the use of pesticides has still been increasing [15]. In addition, a previous study has indicated that there is a frequent appearance of pesticide biomarkers in urine among pregnant women in rural Bangladesh [16]. Thus, pesticide poisoning is a major problem in rural Bangladesh, and one of the advantages of an interventional study in the area is that it will help prevent pesticide poisoning among farmworkers.

The objective of this study is to investigate the effectiveness of the distribution of the checklist—which summarizes 14 points on pesticide protective behaviors—as an interventional measure for pesticide protection in rural Asia, where pesticide poisoning is a crucial problem.

## 2. Materials and Methods

### 2.1. Study Design and Participants

This study is a community-based interventional study in which a checklist for pesticide protective habits was distributed in rural areas of Narail District in southwest Bangladesh. The Narail district is located in Khulna Division and has an area of 990.23 square kilometers (382.33 sq mi) and a population of 721,668 [17]. 

The field study was conducted in Lohagara Upazila in Narail District. Lohagora Upazila has 12 unions, which is the smallest administrative unit in rural Bangladesh. Lohagara Upazila has a total area of 284.91 square kilometers (110.00 sq mi) [18]. According to the 2011 Bangladesh census, Lohagara Upazila had 51,233 households and a population of 228,594; of these, 11.1% lived in urban areas [19]. The literacy rate was 60.3% compared to the national average of 56.1% [14]. The main crops in these areas are paddy, jute, wheat, mustard, betel leaf, and vegetables, while the main fruits are mango, jackfruit, litchi, banana, papaya, star apple, blackberry, coconut, betel nut, shaddock, and hog-plum. 

For this study, two questionnaire surveys were undertaken just before the distribution of the checklist and 25 days after. The inclusion criteria for the participants were male farmworkers aged between 30 and 60 who had worked on farms for more than five years. The participants were selected from two unions, Laxmipasha and Lohagora, from Lohagora Upazila using the method of convenience sampling. For sample size calculation, we decided that a 20% change in outcome measures could be considered to imply effective intervention, with an alpha error of 0.05 and a power of 0.8. Based on this calculation, 91 participants were required; thus, we decided a sample size as 100 participants with ten percent of margin. Moreover, we considered only male farmworkers as eligible for participation because the majority of farmworkers in the research area were male. At the time of enrolment, the surveyor visited each farmworker’s house one after the other and sought informed consent from each individual. Further, participants who could not read and write were not excluded. 

**Figure 1:**
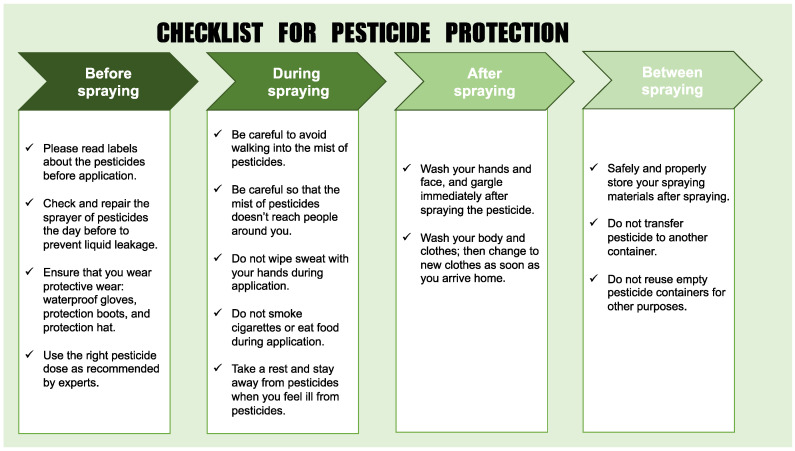
Checklist for pesticide protection.

For the interventional process, the checklist for pesticide protection was first created for the survey using a literature review [7,11]. Next, the validity of the checklist was discussed among health care and pesticide experts from several Asian countries, after which the checklist was developed; in this step, consensus was first obtained among Japanese, Bangladeshi, and Cambodian experts. Finally, the 14 points regarding pesticide protective behavior were summarized under four sections: before, during, after, and between spraying (Figure 1). The checklist was translated into the local language, and local researchers verified the translation. In addition, some pictures that explained the items were inserted into the checklist when the local version was being created. The checklist was printed in color, and all checklists were laminated with a clear cover to prevent it from falling out easily and becoming dirty. This study was approved by the Biosafety, Biosecurity and Ethical Clearance Committee, Jahangirnagar University, Savar, Dhaka, Bangladesh (Reference Number: BBEC, JU/M 2020(12)2). 

### 2.2. Procedure

To identify the effectiveness of the checklist for pesticide-protective habits in rural Bangladesh, a baseline survey was conducted between 19 December 2020 and 12 January 2021. The interview was conducted by the surveyor from the Bridge of Community Development Foundation, a non-government organization registered by the Bangladeshi government. The surveyor was educated on the study design and purpose by SH. The surveyor visited farmworkers’ houses directly to identify eligible participants. After informed consent was obtained, eligible participants were interviewed to gather information on age, sex, years of education, number of years spent working, experience regarding instructions for pesticide use, and symptoms associated with pesticide application. The questionnaire of symptoms was created using a literature review [7]; we adopted multiple-choice questions that allowed the participant to choose all symptoms they experienced during pesticide application and within 24 h. Next, protective behavior during pesticide application was investigated. A questionnaire with nine questions on protective behavior was retrieved from a previous large-scale study in rural China [7]. The frequency of the protective behavior was queried using five levels: always, often, sometimes, rarely, and never.

Immediately after the baseline survey, the surveyor distributed the checklist to the farmworkers and helped them understand each point of the checklist. If the farmworkers could not read or were not clear on the context, then the surveyor provided explanations to ensure that they fully understood each component of the checklist. Additionally, the surveyor also encouraged family members to read the checklist so that they could protect themselves from pesticides as well. Thus, we can assume that all farmworkers were informed and understood the context of the checklist in detail. Here, it is noteworthy that the participants were interested in interventional measures because this attempt was novel. 

The follow-up survey was conducted between 13 January and 7 February 2021. Here, the surveyor asked the same questions regarding protective behavior as the first survey while using the same five frequency levels. 

A scoring system of ten points was applied to the protective behavior frequency (always: 10; often: 6.5; sometimes: 4.5; rare: 2; never: 0). For questions regarding risky rather than protective behavior—such as “Did you prepare pesticides without gloves?”—we applied frequency scores based on inverse protective habits, such as “prepared pesticides with gloves” (See Supplementary Material Table S1 for details). This process was undertaken for all nine behaviors and referred to as “pesticide protective behavioral score”.

### 2.3. Outcomes

The primary outcome was the change in the pesticide protective behavioral scores between the baseline and follow-up surveys, as the frequency score of protective behavior associated with pesticide application. Furthermore, the factors that affect the changes in pesticide protective behavioral scores between the baseline and follow-up surveys were investigated.

### 2.4. Data Analysis

First, social demographics and symptoms of pesticide poisoning during pesticide application and within 24 h were summarized. Second, the median of the “pesticide protective behavioral score” in the baseline and follow-up surveys was determined for each of the nine preventive habits along with its average. Additionally, the median rate of change of the “pesticide protective behavioral score” was determined for each preventive habit along with its average. Paired *t*-test was used to compare the result between pre- and post-survey. Finally, a multivariable linear regression model was used to anticipate the association between the changes in the pesticide-protective behavioral score and social demographics information. The model was selected using Akaike information criterion. A *p*-value less than 0.05 was considered statistically significant. All analysis was performed using STATA IC15 (Lightstone, San Antonio, TX, USA, version 15). 

## 3. Results

A total of 100 eligible males were included in the survey. The median of the years of education was 4.5 years (Table 1). Moreover, 24 participants had experience regarding instructions; of these, the median (interquartile range) of the number of times these instructions were provided was 3 (2, 4). A total of 14 farmworkers had symptoms associated with pesticide application, and the majority of symptoms were neurological, including dizziness, headache, and numbness (Supplementary Material Table S2). 

The median pesticide-protective behavioral score at the baseline survey was zero in five and ten in four of the protective behavioral scores (Table 2). Meanwhile, 25 or 26 days after the distribution of the checklist as an intervention, the median of the pesticide-protective behavioral score increased in five of the protective behavioral scores, which were zero in the baseline survey. In particular, the protective behavior to avoid touching pesticides during application significantly increased in the follow-up survey. The average pesticide-protective behavioral score increased from 4.58 in the baseline survey to 8.11 in the follow-up survey. 

The change in pesticide protective behavioral score was significantly associated with the education year (Table 3). The checklist was more effective in the following groups: those with higher education, who were younger, and who had lower pesticide protective behavioral scores in the baseline survey. On the other hand, symptoms associated with application and differences in union did not affect the rate of change in pesticide protective behavioral scores.

## 4. Discussion

Protecting the health of agricultural workers, as a potentially vulnerable population, is a crucial issue in public health. To achieve this in rural Asia, evidence on easy and effective interventional measures for pesticide protection was essential. The present study aimed to identify the effectiveness of the distribution of a checklist on pesticide protective habits, as an interventional measure for protection from pesticides. 

In this context, this study found that the distribution of a paper checklist on protective behaviors was effective in increasing the frequency of positive pesticide protective habits among farmworkers. More specifically, 25 days after the checklist distribution, the frequency of the five protective behaviors—low in the baseline survey—increased significantly. Many types of checklists on safety or activities regarding pesticide use have been created [20]; however, these had more than 100 items and might not have been suitable for use by farmworkers in rural areas of developing countries. Thus, the present checklist, which only has 14 items and is accessible at all times, was created for this study. Further, this design might be suitable for education and training in this regard in developing countries.

The change in the frequency scores of protective behavior was significantly higher among groups that were highly educated, younger, and that had low levels of protective behaviors in the baseline survey. The adjusted standardized coefficient of the linear regression model for the pesticide protective behavior score was the largest for education as an explanatory variable. The effectiveness of the checklist was higher in the younger age group and in the higher educational group. Thus, a different method of intervention might be more effective for groups with lower education and age groups with lower proportions of literacy.

In the baseline survey, the frequency scores of protective behavior were clearly divided into two groups: frequently or rarely undertaken. Further, the results on the frequency of protective behavior were different from those in previous studies in rural China, indicating that protective behaviors vary between regions [7]. Moreover, behaviors, which had been always performed, did not have to be included in the checklist because further improvement would be impossible. Thus, investigations on the type of protective behaviors that are neglected in each area should be conducted before the development of a checklist. Additionally, neglected protective behavior should be included in local checklists, since there are many pesticide-protective behaviors other than the 14 items mentioned here. In this context, the effectiveness of localization of checklists is also associated with the World Health Organization (WHO) surgical safety checklist [21], and such ingenuity might be required.

When interpreting the results of the present study, several limitations should be considered. First, the primary outcome was set as a subjective report on the frequency of behavior and is not an objective outcome. Second, the present study did not use control groups. Third, the sampling method used was convenience sampling in each union. Fourth, the duration of the survey interval was short; consequently, we did not investigate the long-term effects of pesticide use. Fifth, the outcome values of the multivariate linear regression model were calculated as the sum of each set of nine pesticide-protective scores, but its validity was not investigated. Further, the names of the pesticides that caused the symptoms were not obtained. Additionally, the median of four scores was ten at pre-survey; thus, we could not effectively see the changes in these four scores. Finally, a validation study on the checklist was not performed before the survey. However, despite these limitations, the present study was the first study that investigated the effectiveness of an easy paper checklist on protective behaviors from pesticides in rural Bangladesh.

## 5. Conclusions

The distribution of a paper checklist on pesticide-protective behaviors could be effective in increasing the frequency of positive pesticide-protective behaviors among farmworkers. These intervention measures should be implemented to increase knowledge and awareness of such habits in a manner that is flexible and considers the local population for the sake of protecting the health of farmworkers—a potentially vulnerable population. 

## Figures and Tables

**Table 1:** Participant characteristics.

	Number
Age (median (IR))	41.5 (35, 56)
Years of education (median (IR))	4.5 (0, 8)
Years spent working (median (IR))	24 (17.5, 38)
Address Union	
Laxmipasha	58
Lohagara	42
Experience with instructions	
yes	24
no	76
Number of symptoms	
0	86
1	6
2	4
3	4
Duration of survey interval	
25 days	74
26 days	26

IR = interquartile range.

**Table 2:** Frequency scores of protective behaviors during pesticide application in each survey (10 = always, 0 = never).

Score Name	Baseline Survey Median (IR)	Follow-Up Survey Median (IR)	Changing Rate Median (IR)
Read label	0 (0, 0)	4.5 (0, 6.5)	4.5 (0, 6.5) *
Prepare using gloves	0 (0, 0)	5.5 (5.5, 8)	5.5 (5.5, 8) *
Use protecting equipment	0 (0, 0)	6.5 (6.5, 10)	6.5 (6.5, 10) *
Avoid eating with application	10 (10, 10)	10 (10, 10)	0 (0, 0)
Avoid wiping sweat	0 (0. 5.5)	10 (10, 10)	7.25 (4.5, 10) *
Avoid leaking	10 (10, 10)	10 (10, 10)	0 (0, 0) *
Avoid physical contact	0 (0, 2)	6.5 (6.5, 6.5)	6.5 (4.5, 6.5) *
Take a rest when feeling ill	10 (10, 10)	10 (10, 10)	0 (0, 0) *
Take a shower	10 (10, 10)	10 (10, 10)	0 (0, 0)
**Average**	4.58 (4.44, 5.28)	8.11 (7.61, 9.00)	3.44 (2.72, 3.92) *

IR = interquartile range, * = *p*-value < 0.01 with paired *t* test.

**Table 3:** Liner regression model (adjusted standardized coefficient) for changes in pesticide protective behavioral scores.

	B	95% CI for B	β	*p*-Value
Age (year)	−0.02	−0.03, −0.01	−0.29	<0.001
Education (year)	0.12	0.08, 0.16	0.43	<0.001
Symptoms (ref. no)				
yes	−0.28	−0.63, 0.07	−0.10	0.114
Address (ref. Laxmipasha)				
Lohagara	0.17	−0.07, 0.42	0.09	0.160
Protective score in the first survey	−0.67	−0.87, −0.47	−0.40	<0.001

B = unstandardized regression coefficient, CI = confidential intervals, β = standardized coefficient, Adjusted R^2^, 0.6653.

## Data Availability

Data are available upon request to the corresponding author.

## Supplementary Materials

The following are available online at https://www.mdpi.com/article/10.3390/ijerph18179349/s1, Table S1: Frequency scores of protective behaviors during pesticide application, Table S2: Symptoms associated with pesticide application.
