# Supplementary material for: The Increase in Frequency of Protective Behavior against Pesticide Poisoning in Narail, Bangladesh through Use of an Easy Paper Checklist; an Interventional Study
Source: Int J Environ Res Public Health. 2021 Sep 4;18(17):9349. doi: 10.3390/ijerph18179349 (PMC8431624; doi:10.3390/ijerph18179349)
Supplement: Supplementary file 1 [file ijerph-18-09349-s001.zip › ijerph-1361955-SI.pdf]

Supplemental Table S1. Frequency scores of protective behaviors during pesticide application (10 = always, 0 = never).

Score name	Questions	always	often	sometimes	rare	never
Read label	Did you read labels about the pesticides before application?	10	6.5	4.5	2	0
Prepare using gloves	Did you prepare pesticides without gloves?	0	3.5	5.5	8	10
Use protecting equipment	Did you use personal protecting equipment/clothing during application?	10	6.5	4.5	2	0
Avoid eating during application	Did you smoke cigarettes or eat food during application?	0	3.5	5.5	8	10
Avoid wiping sweat	Did you wipe sweat with your hands during application?	0	3.5	5.5	8	10
Avoid leaking	Was your knapsack leaking during application?	0	3.5	5.5	8	10
Avoid physical contact	Did you avoid physical contact with liquid pesticides during application?	10	6.5	4.5	2	0
Take a rest when feeling ill	Did you continue to work when you felt ill from pesticides?	0	3.5	5.5	8	10
Take a shower	Did you take a shower after pesticide application?	10	6.5	4.5	2	0

Supplemental Table S2. Symptoms associated with pesticide application.

	Number
Dizziness	7
Headache	5
Numbness	1
Vomiting and nausea	1
Thirst	1
Dyspnea/short breath	1
Eye problems	1
Arrhythmia or tachycardia	1
